# An Unusual Case of Injury of Thorax

**Published:** 1912-06

**Authors:** George Foy

**Affiliations:** Fellow of the Royal Society of Medicine, London. Dublin


### An Unusual Case of Injury of Thorax.

GEORGE FOY, M. D., F. R. Cl.
Fellow of the Royal Society of Medicine, London.
Dublin

THE BUFFALO MEDICAL JOURNAL for October, 1895, opens with a very interesting article on "Bruises of the Lung" by Dr. John Parmenter. He deals with cases in which the thoracic walls remain intact; "that is to say, no wound of the thoracic walls together with fracture or dislocation of the ribs; cases, therefore, in which there is no open communication between the point of impact of the acting force and the lesion in the lung."

The article is of value in that it drew attention, like Sir Thomas Browne's "Pandoria" to a popular error, to wit: that weighty bodies cannot pass over the thoracic walls without fracturing the osseous walls of the cavity, and showed that laceration of lung tissue may result without injury to the chest walls. There are some sixteen or seventeen such cases recorded. The majority of the injured were, as might be inferred, children; the youngest of which was that reported by Dr. Wicks, Guy's Hospital Reports for 1860, of a child seven years old, and the eldest a man forty years. To these cases I would add the following one which is remarkable in that neither the thoracic walls nor the thoracic viscera were injured. My patient, a healthy, chubby, strong boy, 10 years old, was on December 21, 1911, crossing one of our city streets when a passing dray cart knocked him down. The boy fell on his face, losing two front teeth from the fall, and the left wheel of the dray passed over the thorax, leaving a broad bruised line from left to right across the scapula. Three men, the boy's father and two laborers, were present at the time of the accident and one of them lifted the lad from the ground. The boy was terribly frightened, unconscious, and bleeding from his mouth. When first examined the case looked very serious; but it was soon found that the bleeding was due to the knocking out of the teeth, and as the boy gradually recovered consciousness and had no fracture, emphysema, nor any evidence of concussion, he was sent home from hospital; where he came under the care of the family medical attendant. He very naturally concluded that the wheel of the dray, which weighed 6½ cwt., could not have passed over the child's chest; but the broad bruised mark on the back of the thorax and the torn clothes ultimately convinced him that the witnesses to the accident were correct when they said "the dray wheel went over the child's body as he lay on his face on the ground."

I may add that the dray was on good springs and the driver was sitting on the shaft of the dray on the side opposite to that under which the child fell. Thirty-four days after the accident a careful physical examination could find no trace of any injury and the boy returned to school, where he shows no trace of cerebral injury for he retains his position as head of his class. I may add that the accident gave rise to a law-suit in which the facts as stated above were proved in Court.

